# Effects of attentional bias modification on chronic low back pain in older outpatients: A randomized crossover trial (pilot study)

**DOI:** 10.1097/MD.0000000000027738

**Published:** 2021-11-12

**Authors:** Takashi Hasegawa, Keita Nishi, Akira Nakashima, Takefumi Moriuchi, Naoki Iso, Hironobu Koseki, Takayuki Tabira, Toshio Higashi

**Affiliations:** a Wajinkai Medical Corporation, Wajinkai Hospital, Nagasaki, Japan; b Unit of Rehabilitation Sciences, Graduate School of Biomedical Sciences, Nagasaki University, Nagasaki, Japan; c Macroscopic Anatomy, Graduate School of Biomedical Sciences, Nagasaki University, Nagasaki, Japan; d Unit of Rehabilitation Sciences, Tokyo Kasei University, Saitama, Japan; e School of Health Sciences, Faculty of Medicine, Kagoshima University, Kagoshima, Japan.

**Keywords:** attentional bias, chronic pain, low back pain

## Abstract

**Objectives:**

In the present study, the effect of attentional bias modification (ABM) on older outpatients, with chronic low back pain, was examined.

**Design:**

This was a single-center, randomized, single-blinded, crossover trial and patients were randomly divided in a 1:1 allocation ratio into two groups: an ABM Leading group and an ABM Trailing group.

**Participants:**

Forty-three outpatients with chronic low back pain participated.

**Interventions:**

Patients were evaluated four times and the treatments were ABM + Normal intervention or Normal intervention only.

**Outcomes:**

Outcome measures included pain intensity on the Numerical Rating Scale, the Pain Catastrophizing Scale, Fear-Avoidance Beliefs Questionnaire, Hospital Anxiety and Depression Scale, Somatic Symptom Scale-8, and EuroQol 5 Dimension-3 levels questionnaire. In addition, we performed the 30-second Chair-Stand test and the Timed Up & Go test for physical function evaluations.

**Results:**

There was no change in pain intensity due to ABM. However, the total Pain Catastrophizing Scale score was significantly decreased, and the EuroQol 5 Dimension-3 levels questionnaire and 30-second chair-stand test were significantly improved (*P* <.05).

**Trial registration:**

The Health Science Ethics Committee, Graduate School of Biomedical Sciences, Nagasaki University (permit number: 17060861), and the clinical trial was registered with UMIN (UMIN000029424).

## 1 Introduction

Pain is defined by the International Association for the Study of Pain as “an unpleasant sensory and emotional experience associated with actual or potential tissue damage or described in terms of such damage.”^[1]^ Pain is broadly classified into two types namely an “acute pain” which lasts for up to 6 weeks triggered by noxious stimuli, and a “chronic pain” which lasts longer than 3 months.^[2]^

The prevalence of chronic pain ranges from as high as 54.4% to 78.8% in persons over 65 years of age.^[3–5]^ The world population is rapidly aging and the number of individuals over 65 years are expected to reach 1.5 billion by 2050.^[6]^ Therefore, the number of people with chronic pain are likely to increase in the future. The incidence of chronic pain has been reported to be high in the neck (15.34%), lower back (27.18%), and knee (29.97%).^[7]^ Additionally, patients with chronic pain show a low health-related quality of life index, high psychological distress,^[8]^ decreased mobility,^[9]^ and physical inactivity.^[10]^ For these reasons, there is an urgent need to consider a new remedy approach to chronic pain, especially for the older people.

In psychological models of chronic pain, focusing on pain is a central element.^[11–13]^ In particular, the tendency to select information related to pain over painless information is associated with an increased pain severity, fear and catastrophic thinking, motivation to avoid painful activities, and increasing disability.^[14,15]^ Cognitive behavioral therapy (CBT)^[16]^ is widely known as a treatment for the psychosocial aspects of chronic pain that identifies maladaptive thoughts and behaviors, corrects them, and encourages behavioral changes.^[17]^ The effects on chronic low back pain have been reported as a decrease in pain intensity, less activity avoidance due to pain,^[18]^ and less catastrophic thinking about pain.^[19]^

However, in order to implement CBT, therapists must undergo rigorous training and obtain appropriate experience, as the effectiveness of CBT can depend on the skill of the therapist. In recent years, attentional bias modification (ABM),^[20]^ which addresses the psychological aspects of chronic pain, has attracted attention. The method displays language or facial expressions on a computer (threat stimulus and neutral stimulus) and corrects the bias of attention caused by the emotional value of the stimulus by selecting a neutral stimulus. ABM is a treatment aimed at correcting and decreasing the pain. Sharpe et al reported that using ABM for 3 months in patients with chronic pain (average 45.6 ± 14.54 years) for an average pain duration of 110 months (approximately 9 years) improved the bias in attention to pain.^[21]^ In recent years, ABM with a 4-week intervention period has been attracting attention as a promising new treatment for psychological problems such as anxiety disorders.^[22,23]^ The advantage of ABM is that the existing programs can be used on a computer and even beginners can use them relatively easily. Recently, programs on smartphones have also been developed so that ABM can be used for self-training.^[24]^ Shiasy et al reported the effect of combining ABM and transcranial direct current stimulation (tDCS) on chronic low back pain in 2020.^[25]^ The report indicated that five sessions of ABM and ABM + tDCS could reduce pain-related psychological consequences significantly, compared to the control and sham tDCS groups. However, majority of studies have focused on young people, and there are few reports on people over 65 years of age.

For these reasons, we aimed to examine the intervention effect of ABM on outpatients with chronic low back pain from both psychological and physical function perspectives. We hypothesized that ABM would be useful in reducing chronic low back pain.

## 2 Methods

### 2.1 Study protocol design

This study adopted a single-center, randomized, crossover design and the procedure outlined by the CONSORT 2010^[26]^ statement. Random numbers were generated by a computer and patients were randomly assigned at a 1:1 allocation ratio into one of two groups: an ABM Leading group and an ABM Trailing group. The sample size was calculated using a G-power (statistical power analyses for Windows and Mac, version 3.1.9.2) and a total of 36 participants obtained. The program created a random assignment sequence and registered participants.

**Figure 1:**
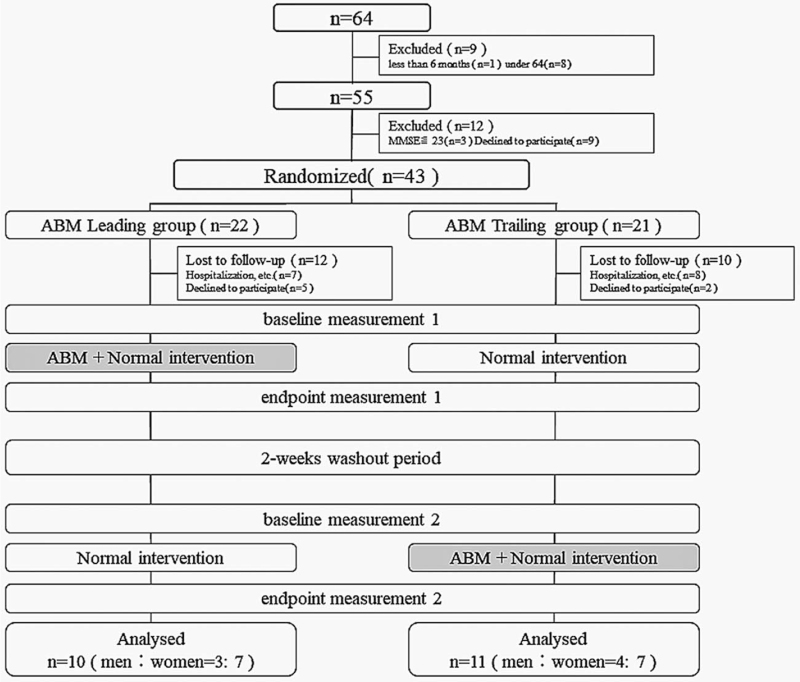
Flow diagram of the study. ABM = attentional bias modification.

There were no placebo interventions and no blinding of assignments to patients. Evaluators with >4 years of experience conducted the pre-evaluation training and were blinded to group assignments. Each intervention period of ABM + Normal intervention and Normal intervention was 12 weeks. Four evaluations were performed before and after the intervention period for each patient. Crossovers trial were performed with a 2-week washout period between interventions, based on previous pain intervention studies.^[27,28]^ The intervention period and washout period ended after approximately 26 weeks in total. The study procedure is summarized in Figure 1. This study was approved by the Health Science Ethics Committee, Graduate School of Biomedical Sciences, Nagasaki University (permit number: 17060861), and the clinical trial was registered with UMIN (UMIN000029424).

### 2.2 Patients and procedures; ABM training

Patients in this study were older outpatients undergoing rehabilitation at Wajinkai Hospital. The criteria for inclusion were: (i) chronic lower back pain for ≥6 months, (ii) aged over 65 years old, and (iii) independence in activities of daily life. The criteria for exclusion were: (i) suspected cognitive decline (Mini-Mental State Examination 23 points or less), (ii) receiving individual physical therapy sessions, and (iii) no consent to participate. Patients who met the selection criteria were notified about the aim of the research and completed the consent form. The study period was from October 1, 2017 to December 31, 2018.

We used ABM Trainer for Windows (ideoquest, Tokyo, Japan) to randomly display images (human expressions) on the top and bottom of a computer screen. We displayed threat stimuli (angry/fear) to cause negative emotions, and neutral stimuli (no facial expression) to cause neutral emotions, and patients were instructed to select the neutral stimulus as quickly as possible. Patients were asked to press the corresponding up/down button after the “E” display appeared. Images used were facial expressions with emotional values identified with reference to the Japanese Female Facial Expression. A total of 128 pairs of images were presented randomly each time, and the training time was approximately 10 minutes, and patients sat in a room separate from the rater to perform the task. The intervention period was 12 weeks, twice a week, for a total of 24 sessions. ABM stimuli are language-based and facial expression-based. Previous studies have reported that language-based trials are more useful.^[20,29]^ However, in order to eliminate the influence of the characteristics of the patients’ native language, this study adopted facial expression-based stimuli.

### 2.3 Outcome measurements and primary outcome

The measurement points before and after the intervention and before the washout period were set as baseline measurement 1 and endpoint measurement 1, respectively, and the measurement points before and after the intervention and after the washout period were set as baseline measurement 2 and endpoint measurement 2, respectively. Primary and secondary outcomes were measured at each measurement point. The primary outcome, current pain intensity, was assessed using an 11-point numerical rating scale (NRS). NRS is the most commonly used pain assessment tool^[30]^ and it is rated as an 11-point scale where 0 = “no pain” and 10 = “pain as bad as you can imagine” or “worst pain imaginable”.^[31]^

### 2.4 Secondary outcomes

#### 2.4.1 Pain catastrophizing scale

The Pain Catastrophizing Scale (PCS) ^[32]^ was used to evaluate the psychological aspects of pain. It is a 13-item questionnaire, with each item evaluated using five categories on a Likert scale where “0 = not applicable at all” and 4 = “very applicable”. It consists of three sub-items: “Rebellion”, “Helplessness”, and “Expansion”. The higher the total score, the stronger the catastrophic thinking.

#### 2.4.2 Fear-avoidance beliefs questionnaire

The Japanese version of the Fear-Avoidance Beliefs Questionnaire (FABQ-J) was used to evaluate avoidance thinking that could be caused by back pain.^[33]^ FABQ-J is a five-item questionnaire that uses seven categories on a Likert scale where 0 = “not at all” and 6 = “exactly.” Strong fear-avoidance thinking was defined by a score of ≥15 points.

#### 2.4.3 Hospital anxiety and depression scale

The Hospital Anxiety and Depression Scale (HADS) was used to assess the mental state of anxiety and depression in patients in the previous week.^[34]^ HADS consists of 14 items, including seven anxiety items and seven depression items. There are four possible responses on a Likert scale where 1 = consent and 4 = denial, and the corresponding score was subtracted from 28 points (very strong anxiety and depression) for each evaluation.

#### 2.4.4 Somatic symptom scale-8

We used the Somatic Symptom Scale-8 to assess the burden of physical symptoms.^[35]^ There are eight questions to assess the physical symptoms of the previous week. There are five possible answers on a Likert scale where 0 = “not frustrating” and 4 = “very frustrating”.

#### 2.4.5 EuroQol 5 dimension

The EuroQol 5 Dimension-3 levels questionnaire **(**EQ-5D-3L) was used to assess health-related quality of life as the relationship with chronic pain has been reported.^[36,37]^ This questionnaire consists of five multiple-choice items.

#### 2.4.6 30-second chair-stand test

The 30-second Chair-Stand test (CS-30) was used to evaluate lower extremity muscle strength. The CS-30 was developed for the purpose of evaluating lower limb strength in older people.^[38]^ It determines how many times the patient can rise from a chair in 30 seconds without using the upper limbs. After the demonstration, a practice trial was given, followed by a 30-second test trial. The CS-30 has been reported to be associated with multiple factors such as lower limb strength and balance function.^[39]^

#### 2.4.7 Timed Up & Go test

The Timed Up & Go test^[40]^ measured the time taken to stand up from a 46 cm-high chair, turn at a mark 3 m ahead, and sit down again. After the demonstration, one practice trial was given, followed by one test trial.

### 2.5 Statistical analysis

The final results only included patients who completed all measurements. The normality of the distribution was tested with Shapiro-Wilk analysis. Two baseline values were compared using the paired sample t-test for the parameters with normal distributions and the Wilcoxon signed-rank test for the parameters without normal distribution. For descriptive statistics, the data were expressed as mean ± SD, and the significance level was set at 5%. We estimated that a 2-week washout period was enough and found a lack of significance (*P* > .05). The differences between the two baseline and endpoints measurements were calculated, and the parameters for the ABM period (ABM) and non-ABM period (Non-ABM) were compared. SPSS for Windows (SPSS version 22.0, IBM, Armonk, NY) was used for all analyses. We report Cohen's d as effect size estimates.

## 3 Results

### 3.1 Patient characteristics

Fifty-five patients met the inclusion criteria. Three patients with an Mini-Mental State Examination of fewer than 23 points and nine patients who did not provide consent were excluded. The remaining 43 patients were randomly assigned to an ABM Leading group (n = 22) or an ABM Trailing group (n = 21).

There were seven hospitalizations and five consent withdrawals in the Leading group, and eight hospitalizations and two consent withdrawals in the Trailing group. There were 10 participants in the ABM leading group (77.9 ± 7.0 years, male: female ratio = 3: 7) and 11 in the ABM trailing group (79.9 ± 5.9 years, male: female ratio = 4: 7) who completed the endpoint measurement 2 (Table 1) (Fig. 1). There were no significant differences in the two baselines (baseline measurement 1, baseline measurement 2) of activities of daily life ability and pain intensity, psychological, physical function between the two groups (Table 2).

**Table 1:** Clinical characteristics.

	ABM leading group	ABM trailing group
mean age, years (SD)	77.9 (7.0)	79.9 (5.9)
gender (%)		
male	30.0	36.4
female	70.0	64.6
primary pain diagnosis (%)		
degenerative lumbar spondylosis	20.0	72.7
lumbar spinal stenosis	20.0	18.2
lumbar spondylolisthesis	0.0	9.1
medications taken during the trials (%)^∗^		
Pregabalin	20.0	27.3
NSAIDs	20.0	18.2
Acetaminophen	10.0	36.4
Ketoprofen patch	30.0	9.1
None	30.0	27.3

∗ There were duplicate answers. ABM = attentional bias modification, NSAIDs = non-steroidal anti-inflammatory drugs, SD = standard deviation.

**Table 2:** Comparison between the ABM Leading group and the ABM Trailing group.

		baseline measurement 1				baseline measurement 2			
		ABM Leading group	ABM Trailing group	*P* value^∗^	Effect sizes (Cohen's d)	ABM Leading group	ABM Trailing group	*P* value^∗^	Effect sizes (Cohen's d)
ADL	BI	97.5 (3.4)	94.2 (6.4)	.16	.31	97.0 (4.0)	94.1 (6.4)	.27	.25
pain intensity	NRS	4.7 (1.4)	5.7 (1.4)	.12	.34	4.4 (1.3)	5.6 (1.9)	.13	.33
psychological measures	FABQ-J	13.9 (6.5)	17.2 (5.9)	.34	.21	13.7 (5.9)	17.6 (5.6)	.23	.26
	PCS total	28.4 (11.1)	28.6 (7.9)	.57	.21	28.0 (10.3)	28.2 (6.9)	.70	.09
	Rumination	12.1 (5.0)	11.2 (3.3)	.67	.09	11.1 (5.1)	9.7 (3.0)	.92	.02
	Helplessness	10.2 (5.5)	9.7 (3.7)	.89	.03	10.5 (4.1)	9.8 (2.1)	.67	.09
	Magnification	6.1 (2.8)	7.6 (3.0)	.34	.21	6.4 (2.6)	8.6 (3.3)	.14	.33
	HADS (A)	6.7 (2.3)	6.4 (1.8)	.86	.04	6.7 (1.7)	6.3 (1.3)	.56	.13
	HADS (D)	7.4 (2.2)	6.6 (3.5)	.37	.20	7.0 (2.5)	6.6 (2.9)	.67	.09
	SSS-8	11.6 (4.9)	13.4 (5.5)	.57	.12	10.9 (4.2)	12.7 (5.2)	.55	.13
	EQ-5D-3L	0.62 (0.17)	0.69 (0.18)	.91	.03	0.66 (0.12)	0.64 (0.13)	.94	.02
physical measures	CS-30	11.9 (5.1)	10.5 (5.3)	.50	.15	12.5 (5.2)	9.9 (4.9)	.17	.30
	TUG	11.3 (3.2)	13.0 (4.6)	.48	.15	11.6 (3.3)	13.6 (4.3)	.23	.26

Data are presented as mean (standard deviation). ABM = attentional bias modification, ADL = activities of daily living, BI = Barthel Index, CS = 30-second chair-stand test, EQ-5D-3L = EuroQol 5 Dimension-3 levels questionnaire, FABQ-J = the Japanese version of the Fear-Avoidance Beliefs Questionnaire, HADS = Hospital Anxiety and Depression Scale, NRS = numerical rating scale, PCS = Pain Catastrophizing Scale, SSS-8 = Somatic Symptomatic Scale 8, TUG = Timed Up & Go test.

∗ Mann-Whitney *U* test.

### 3.2 Pain and psychological measures

The NRS scores were -0.57 ± 1.37 in the ABM trials and -0.05 ± 0.65 in the Non-ABM trials (*P* = .19; Cohen's d = .29). On the other hand, total PCS scores showed a significantly greater decrease in the ABM trials (ABM: -1.86 ± 5.97 vs Non-ABM: -0.38 ± 4.50; *P* = .01; Cohen's d = .55). In addition, there were no significant differences in the subordinate PCS items. EQ-5D-3L showed significantly greater scores in the ABM trials (ABM: 0.07 ± 0.10 vs Non-ABM: 0.03 ± 0.09; p < .01; Cohen's d = .63). Furthermore, there were no significant differences observed between the intervention and non-intervention trials for FABQ-J, HADS (A), HADS (D), and somatic symptom scale-8 (Table 3).

**Table 3:** Comparison of psychological evaluation between the ABM and non-ABM groups.

		ABM	Non-ABM	*P* value^∗^	Effect sizes (Cohen's d)
pain intensity	NRS	−0.57 (1.37)	−0.05 (0.65)	.19	.29
psychological measures	FABQ	−0.71 (2.81)	−0.52 (3.33)	.63	.11
	PCS total	−1.86 (5.97)	−0.38 (4.50)	.01^†^	.55
	Rumination	−1.71 (2.51)	−0.67 (2.95)	.11	.35
	Helplessness	−0.05 (2.79)	0.00 (2.47)	.7	.08
	Magnification	−0.10 (1.74)	0.29 (1.08)	.19	.28
	HADS (A)	−0.14 (1.58)	−0.19 (2.06)	.89	.03
	HADS (D)	−1.00 (1.54)	−0.57 (1.47)	.18	.29
	SSS-8	−1.00 (2.45)	−0.86 (2.92)	.63	.11
	EQ-5D	0.07 (0.10)	0.03 (0.09)	<.01^†^	.63

Data are presented as mean (standard deviation). ABM = attentional bias modification, EQ-5D-3L = EuroQol 5 Dimension-3 levels questionnaire, FABQ-J = Fear-Avoidance Beliefs Questionnaire, HADS = Hospital Anxiety and Depression Scale, NRS = numerical rating scale, PCS = Pain Catastrophizing Scale, SSS-8 = Somatic Symptomatic Scale 8

∗ Wilcoxon signed-rank test.

† statistically significant (*P* < .05).

### 3.3 Physical function measures

Changes in CS-30 scores were 2.05 ± 2.01 in the ABM trials and 0.19 ± 1.43 in the Non-ABM trials and this difference was significant (*P* < .01; Cohen's d = .77). However, there was no significant difference regarding Timed up & Go test (Table 4).

**Table 4:** Comparison of physical function evaluation between the ABM and non-ABM groups.

		ABM	Non-ABM	*P* value^∗^	Effect sizes (Cohen's d)
Physical function	CS-30	2.05 (2.01)	0.19 (1.43)	<.01^†^	.77
	TUG	−0.18 (1.28)	0.20 (0.92)	.09	.37

Data are presented as mean (standard deviation).

∗ Wilcoxon signed-rank test.

† statistically significant (*P* < .05). ABM = attentional bias modification, CS-30 = 30-second chair stand test, TUG = Timed Up & Go test.

## 4 Discussion

In this study, we examined the effect of an ABM intervention on older outpatients with chronic low back pain using a randomized controlled trial design. There was no significant difference in the intensity of pain before and after the intervention. In a recent ABM report, 20 pairs of pain-related terms combined with irrelevant terms were not associated with pain intensity in a trial of cold pressure stimulation versus simulated training.^[41]^ Furthermore, in a study of the effect of home-based self-training using ABM for 4 weeks for chronic pain, there was no difference between the placebo group and the non-training group.^[42]^ In this respect, the results were the same as this study, but further examination is necessary because the types of pain and the attributes of the patients were different between studies.

On the other hand, a significant decline was observed in total PCS score, suggesting the possibility that ABM had a positive effect on catastrophic thinking. The means of directing attention to pain-related stimuli varies among individuals, but the greater the fear of pain, the more likely it is to be induced by negative stimuli.^[43]^ In previous studies using eye trackers, increasing PCS has been reported to increase attention bias for pain.^[44]^ By correcting for the negative attention bias, a general improvement in catastrophic thinking about pain is obtained. However, in the present study, we did not reveal any significant differences in the subordinate items, and it was not possible in this study to determine the factors involved in catastrophic thinking about pain.

Furthermore, there was a significant improvement in CS-30 as a measure of physical function. In general, improvements in CS-30 scores means improvement of muscle strength and posture balance, and there are reports that it is useful as an auxiliary test for early diagnosis of preventative care in older people.^[45]^ However, it is unlikely that muscular strength directly increased considering the characteristics of patients in this study. Therefore, we believe that the improvement in PCS had some positive effects on the fear of exercise and may have led to an improvement in performance. We believe that the psychological factors associated with improving PCS and CS-30 are due to improvements in EQ-5D-3L. Regarding the effects of changes in psychological aspects on physical function, some studies have shown that improvements in depression tended to contribute to improved grip strength,^[46]^ and conversely, changes in PCS did not contribute to the degree of physical disability.^[47]^ Currently, a consistent view has not been obtained.

This study has several limitations. First, because the study was conducted in a single facility, the sample size was small, lumbar diseases were grouped, and disease-specific validation was not possible. In this study, the pre-calculated sample size of 36 participants could not be reached. In the future, we would like to plan a larger multicenter randomized controlled trial to increase the number of participants and examine the effect of ABM on each lumbar disease groups. Second, the procedure used in this study was the same as that used for young people in previous studies. Bowler et al mentioned that with regard to the appropriate image presentation time, the speed of the response is different in young and older people, and this may have affected the attention to the task.^[48]^ Third, nearly 50% of the participants dropped out of this study. It is thought that this was due to the fact that some of the older people were unfamiliar with PC operation. In addition, the older outpatients were more likely to get sick and discontinue the study than healthy people, and many participants were hospitalized during the intervention period. Finally, there was no placebo group and the participants could not be blinded.

In conclusion, the results suggest that ABM for older outpatients with chronic low back pain may contribute to improved psychological and physical function; however, it may have no effect on pain intensity. In the clinical setting, pre-exercise ABM training for patients with chronic low back pain may play a role in mental conditioning (such as reducing exercise fear) and help improve performance.

## Acknowledgments

The authors would like to thank all the patients who participated in this study. They would also like to thank Editage (www.editage.jp) for English language editing.

## Author contributions

**Data curation:** Takashi Hasegawa.

**Formal analysis:** Keita Nishi, Akira Nakashima, Takefumi Moriuchi, Naoki Iso.

**Project administration:** Toshio Higashi.

**Supervision:** Hironobu Koseki, Takayuki Tabira, Toshio Higashi.

**Writing – original draft:** Takashi Hasegawa.

**Writing – review & editing:** Hironobu Koseki, Takayuki Tabira, Toshio Higashi.
